# Individual and collective foraging in autonomous search agents with human intervention

**DOI:** 10.1038/s41598-021-87717-7

**Published:** 2021-04-19

**Authors:** Daniel S. Schloesser, Derek Hollenbeck, Christopher T. Kello

**Affiliations:** 1 Cognitive and Information Sciences, University of California, Merced, USA; 2 Mechanical Engineering, University of California, Merced, USA

**Keywords:** Psychology, Human behaviour, Behavioural ecology

## Abstract

Humans and other complex organisms exhibit intelligent behaviors as individual agents and as groups of coordinated agents. They can switch between independent and collective modes of behavior, and flexible switching can be advantageous for adapting to ongoing changes in conditions. In the present study, we investigated the flexibility between independent and collective modes of behavior in a simulated social foraging task designed to benefit from both modes: distancing among ten foraging agents promoted faster detection of resources, whereas flocking promoted faster consumption. There was a tradeoff between faster detection versus faster consumption, but both factors contributed to foraging success. Results showed that group foraging performance among simulated agents was enhanced by *loose coupling* that balanced distancing and flocking among agents and enabled them to fluidly switch among a variety of groupings. We also examined the effects of more sophisticated cognitive capacities by studying how human players improve performance when they control one of the search agents. Results showed that human intervention further enhanced group performance with loosely coupled agents, and human foragers performed better when coordinating with loosely coupled agents. Humans players adapted their balance of independent versus collective search modes in response to the dynamics of simulated agents, thereby demonstrating the importance of adaptive flexibility in social foraging.

## Introduction

Foraging is oftentimes studied as an activity produced by individual organisms. A single bird flies over an area in search of food, a tiger roams the jungle, or a person scans their terrain for resources. Other times, foraging is studied as a collective activity that groups of organisms engage in^1–4^. Collective foraging occurs when groups of organisms interact and move together while searching for resources, and it is more often associated with organisms that have limited cognitive capacities for planning and decision-making. Organisms with greater cognitive capacities may also engage in collective foraging, but they are more likely to exhibit flexibility in switching between different foraging modes depending on various factors. For example, a lion may choose to hunt alone or team up with other lions to find and take down prey^5^. A person may choose to help others harvest a large patch of berries or head off alone in search of unfound patches. Foragers can also communicate information about resources and conditions to each other, and thereby help individuals make decisions about where and with whom to forage^1–4,6,7^.

In the present study, we investigate whether foraging can benefit from the ability to vary between individual versus collective search modes, as a means of improving group foraging performance. A given mode may be more or less advantageous depending on various foraging factors and conditions. For instance, the conditions under which collective foraging is advantageous are often studied through the lens of *social foraging theory*^8^. Group cooperation can outperform individual or independent foraging strategies by finding and exploiting food resources more quickly^9^, and also by providing security from predation among other social benefits^5,10^. However, these benefits may not always be available or sufficiently salient, and they may be outweighed when foraging becomes sufficiently competitive^11^. This dependence on conditions suggests that the ability to modulate between independent and collective modes of foraging may be advantageous for collective foraging performance.

For example, Harel et al. showed that Eurasian griffon vultures (*Gyps fulvus*) switch between independent and cooperative foraging strategies in response to social cues^12,13^. They found evidence that vultures can exchange information about a found carcass even when they are far from its location. Specifically, vultures who previously visited a carcass were more likely to be followed when revisiting the same carcass location. Uninformed vultures apparently used visible cues, such as blood stains on the head and body, to flexibly choose when to collectively forage and with whom. Flexibly switching between these two strategies increased group foraging success by helping uninformed vultures find new carcasses more quickly, and together they were able to consume carcasses more quickly, thereby leaving less chance for other scavengers to share in the meal. Similarly, Geoffroy's spider monkeys (*Ateles geoffroyi*) engage in information transference between naïve and informed foragers which helps to improve their success^14^.

In general, efficient foraging needs to result in both finding as well as consuming enough food for individuals and groups to survive^13,15–17^. For example, Beauchamp (2005) designed a social foraging simulation that demonstrated this principle of efficient foraging indirectly. Social foragers consumed food at a faster rate compared with individual foragers, but faster consumption came at the price of consuming less food per individual. There was also reduced variability in the rate of food consumption among social foragers, indicating that social foraging may confer protection against long periods of famine.

The benefits of social foraging have been shown in birds of prey as well^12,18–20^, and the apparent prevalence of collective foraging in nature has led researchers to develop and test formal models of collective foraging, to investigate the underlying principles and processes. For example, Liu and Passino (2004) created a collective foraging model based on balancing forces of attraction and repulsion so that agents tended to position themselves nearby neighbors while keeping some distance. The goal was for agents to find food resources by following gradients to their locations. Coordinating movements with nearby agents was useful for them to collectively follow otherwise unreliable gradients in the simulated environment that led to resources^21–27^.

In the present study, we developed an agent-based simulation in which both individual and collective foraging strategies have their advantages, so being able to vary between them should be advantageous to the group. We created a social foraging simulation in which we could test collections of autonomous agents with movement rules that varied between more and less collective modes of interaction. For our targeted *loose coupling* condition, rules were parameterized to balance individual versus collective search behaviors, we were also able to test how humans move and interact with autonomous foraging agents by including a condition in which a human player controlled one of the search agents. Our aims were to (1) understand how coupling strength in the movements of autonomous agents plays a role in foraging performance by diversifying their collective movement patterns, and (2) test whether human agents with high cognitive capabilities while searching engage in loose coupling with autonomous agents in the service of collective foraging.

Our results showed that balancing attraction and repulsion promoted and sustained coordinated movements because random variability inherent to individual foraging behaviors was averaged out as a result of loose coupling. This balance of attraction and repulsion was accomplished using the so-called *Lennard–Jones potential*^28,29^ applied to govern the degree to which agents are attracted to or repelled from each other as a function of their distance apart.

We manipulated the degree of coupling strength using the Lennard–Jones potential^30^ plus a flocking term that correlated the direction of movement among nearby search agents. We also investigated how the addition of a human agent who can switch between independent and cooperative search strategies affects collective movement patterns and foraging efficacy. We expected the intervention to improve group performance, and we also tested whether the degree of loose coupling, in terms of distancing and flocking among autonomous agents, affects human search performance. By comparing agent-based simulations with and without human intervention, we also investigated the benefits of human memory and decision-making in managing the balance of independent versus collective foraging behaviors.

## Method

### Simulation

The agent-based foraging model was implemented in NetLogo with a 200 × 200 grid of pixels with periodic boundary conditions and based on a previous social foraging model^10^. The grid was empty except for one target at time. The target occupied four pixels and was located at random. The goal was for ten agents to search the task space for a gold star target and find as many of these targets as possible within a set amount of time. Ten agents were chosen to provide enough grouping variability while also allowing human intervention to have a meaningful impact on group coordination and performance. While searching for targets, all agents moved at a constant velocity of 1 pixel per time step. Agents could not “see” the target until they came within a ${d}_{v}=22.5$ pixel radius of their position, which meant the visible area for each agent was 4% of the total game space. With ten agents searching together, it did not take long to find each target, which helped to find multiple targets within minutes of time.

Search was also facilitated by a chaining effect whereby agents could see when other agents in their view had detected the target. The rationale was that agents change their behavior when detecting a target, e.g. they take a more direct path towards a specific point. Other agents may see this behavioral change before they see the target itself, so the behavioral change becomes a target itself to draw an agent to share in the find. This dynamic was implemented by setting a flag on agents who detect the target and adding a rule to drive unflagged agents to converge on a visible flagged agent as they would converge on the target itself.

When agents arrived at target locations, they immediately started consuming the target, one unit of consumption per time unit, where each target consisted of 500 consumption units. Therefore, it required 500 time steps for one agent to fully consume a target. Less time was required to consume as more agents arrived at the target location and consumed it together, simultaneously. Each target disappeared after it was completely consumed, and a new target appeared at a new random location.

Each simulation session was defined by the rules applied to all ten autonomous agents that generated search movements to find each target or agents who found the target (i.e. visual chaining^18^). The default rule present in all conditions was a correlated random walk (CRW), which made each agent wander through space randomly with some tendency to maintain the current heading. Next was a flocking rule that added a tendency for movement in the average direction of nearby agents. Third and final was a distancing rule based on a generalization of the Lennard–Jones potential^28,30^, that drove agents to maintain a given distance from each other. The governing equations for the CRW, flocking, and distancing forces on the $i$-th agent (given $i\ne j$) at time $t$ are as follows:

$${{\mathrm{CRW}}}(\overrightarrow{d}_{N}){\overrightarrow{: d}}_{i,N}=[\mathrm{cos}{({\theta }_{i}(t-\delta t)+ \theta }_{N}),\mathrm{sin}{({\theta }_{i}\left(t-\delta t\right)+\theta }_{N})],$$

$${\mathrm{Flocking }}({\overrightarrow{d}}_{A }): {\overrightarrow {d}}_{i,A}={\sum }_{j=1}^{\Omega }{\overrightarrow{d}}_{j}(t-\delta t),$$

$${{\mathrm{Distancing }({\overrightarrow{d}}_{LJ}): \overrightarrow d}}_{i,LJ}=-{\sum }_{j=1}^{\Delta }\left[{\left(\frac{S}{\Vert {\overrightarrow{d}}_{ij}\Vert }\right)}^{4}-{\left(\frac{S}{\Vert {\overrightarrow{d}}_{ij}\Vert }\right)}^{3}\right]\frac{{\overrightarrow{d}}_{ij}}{\Vert {\overrightarrow{d}}_{ij}\Vert }.$$

For the CRW, ${\theta }_{i}$ represents the current heading of the $i$-th agent and ${\theta }_{N}$ is a correlated random angle given by ${\theta }_{N}=$ ${\theta }_{R}-{\theta }_{L},$ where ${\theta }_{R}\sim U(\mathrm{0,180})$ and ${\theta }_{L}\sim$ *U*(0,180) are independent random turning angles between 0 and 180 degrees. The difference between these random uniform turning angles, produces a symmetric probability distribution from $-180$ to $180$, linearly weighted towards zero. For flocking, the vector ${\overrightarrow{d}}_{j}$ represents the directional heading of the $j$th agent at time $t-\delta t$, where $\delta t$ represents the time step. The flocking direction ${\overrightarrow{d}}_{A}$ is calculated as the sum of the set of all the agents within the vision distance ${d}_{v}$ denoted as $\Omega =\left\{{ \overrightarrow{\mathrm{d}}}_{\mathrm{j}} \right| \Vert {\overrightarrow{d}}_{ij}\Vert <{d}_{v}\}$. The vector ${\overrightarrow{d}}_{ij}$ represents the distance in pixels from the $i$-th and $j$-th agents. The distancing ${\overrightarrow{d}}_{LJ}$ is calculated from the separation parameter $s$ (the desired distance between agents), and the distance ${\overrightarrow{d}}_{ij}$ between pairs of agents belonging to the set $\Delta =\left\{{\overrightarrow{d}}_{ij}\right|$ $\Vert {\overrightarrow{d}}_{ij}\Vert <{1.5d}_{v}\}$. To avoid losing sight of the agents, the separation distance $s$ was set to $s=15$ pixels, which is inside the vision distance ${d}_{v}$. The exponents 4 and 3 in the distancing rule ${\overrightarrow{d}}_{LJ}$ represent the repulsion and attraction terms, respectively. The original exponents values for the Lennard–Jones potential are 12 and 6, respectively. These values were chosen arbitrarily, through trial and error, to reflect the loose coupling between agents. The result is a mostly repulsive force with a weak attractive component that fades away as the agents separate greater than $s$.

**Figure 1:**
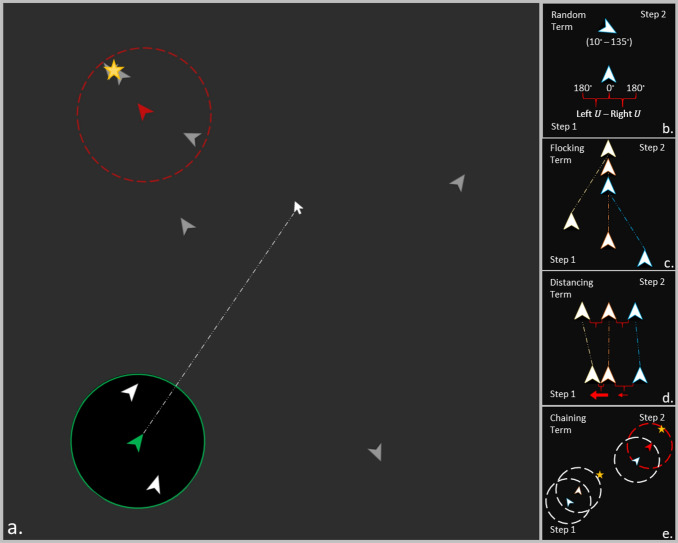
**(a)** Illustrative view of the task space (not shown to players). The green agent and circle represent the human’s agent and their field of view (detailed more below). Area outside their field of view was occluded (greyed out area). The dotted line represents that the human agent moved towards the mouse pointer position, so the human could control movement direction by moving the mouse. **(b)** Random movement shown to be random angular deviations of movement from each previous heading. **(c)** Flocking term directed agents to converge towards a similar shared movement trajectory. **(d)** Distancing term prompted agents to separate from one another when close, and towards each other when further away. **(e)** Visual chaining prompted agents to move directly toward an agent flagged as detecting the target.

**Figure 2:**
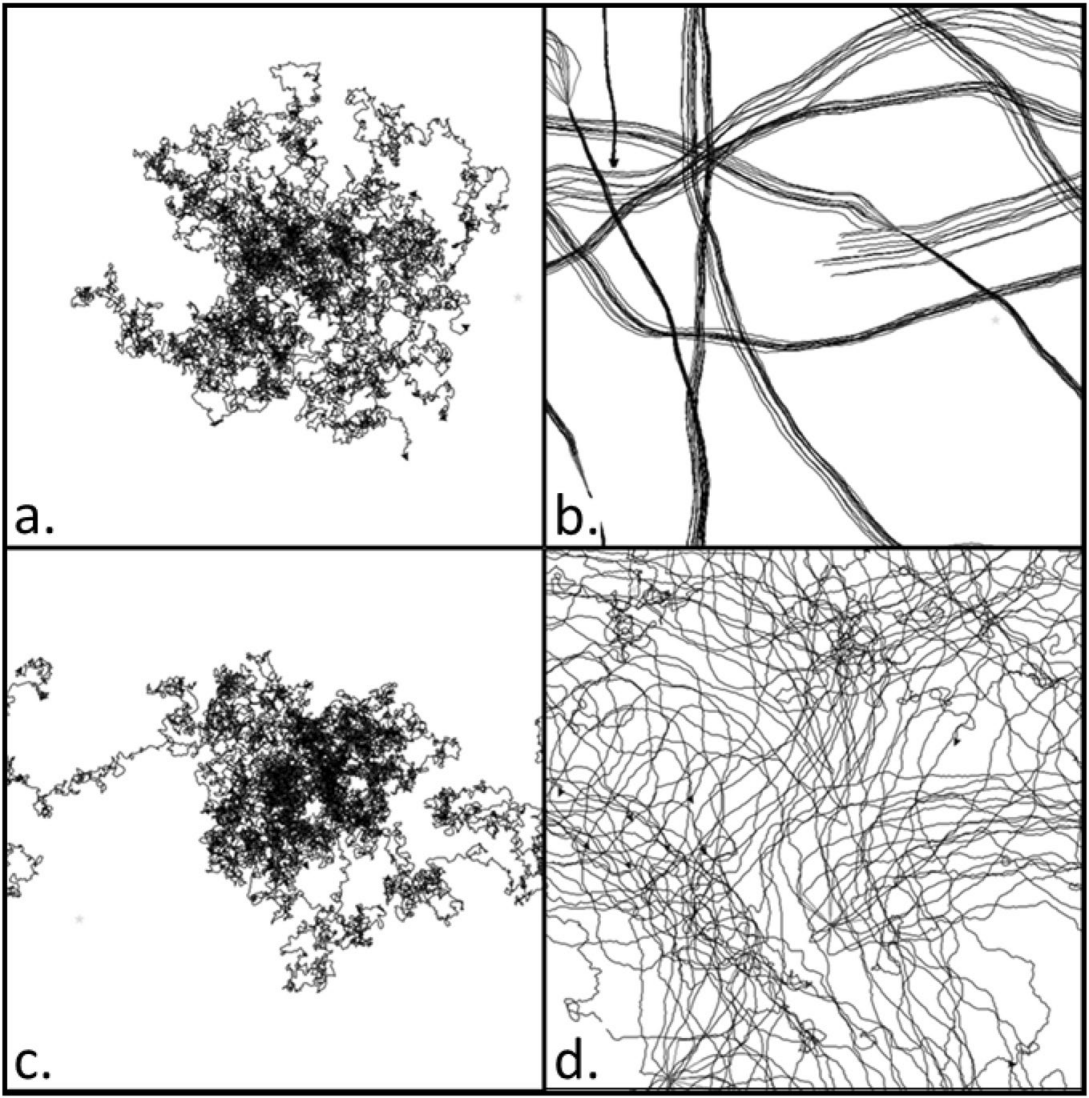
Example movement trajectories for 2000 ticks for each movement condition: **(a)** Random; **(b)** Flocking; **(c)** Distancing; and **(d)** Loose coupling.

The governing equation for the $i$-th agent at time $t$ is found by combining additively, ${\overrightarrow{d}}_{i}={\overrightarrow{d}}_{i,N}+{\overrightarrow{d}}_{i,A }+{\overrightarrow{d}}_{i,LJ}$. Simulation conditions were defined by turning off or on the flocking and distancing rules such that the CRW rule was always in effect, resulting in four different movement conditions: Random (CRW only), Flocking (and CRW), Distancing (and CRW), and Loose Coupling (all three rules combined). See Fig. 1 for visual illustrations of the movement rules and see Fig. 2 for general trajectory examples of each movement condition.

Each simulation session lasted 13,500 game time steps, referred to hereafter as ticks, which corresponded to about eight minutes in real time when simulated through the NetLogo interface. Performance was measured in terms of number of targets found and consumed, which was based on the times needed to find and consume targets. Each movement condition was tested in 60 sessions to match the number of sessions with data collected from participants in the human intervention experiment, described next.

### Participants, materials, and experimental setup

The simulated collective foraging sessions described in the previous section consisted of autonomous agents only, with no human intervention. Matching sessions were conducted with human players, where each session including one human controlling one of the ten agents, as follows.

All methods involving human subjects were carried out in accordance with relevant guidelines and regulations. Additionally, all experimental protocols were approved by the University of California, Merced IRB committee and informed consent was obtained from all subjects prior to participation. All subjects were above the age of 18 years old. Sixty participants from the University of California, Merced were recruited for course credit. After being instructed about the game and filling out the consent form, each participant controlled one of the ten search agents and engaged in collective foraging with the goal of finding and consuming as many targets as possible in the allotted time. Each participant controlled their on-screen avatar by using a computer mouse to place a pointer at the desired location, and then their avatar moved in the direction of the pointer. If the avatar reached the desired location, it kept moving in the same direction until the human player moved the mouse to change course. Participants had the same visual radius as autonomous agents, and their avatar affected autonomous search agent dynamics according to the rules as described above. Autonomous agents did not affect movement of the human avatar except for the chaining rule—the human avatar went straight to an autonomous agent if they were tracking towards the target.

The foraging game was designed to give human players the same operational information and latitude as automated agents, so that the only difference with human players was their memory about prior states of the environment and foraging outcomes, and decision-making based on memory and strategies developed through experience. The simulation ran at about 35 ms per update, and each human player ran through one session of each of the four movements conditions, in counterbalanced order across participants. The number of time steps was chosen to be long enough to elicit variability in performance, but short enough to run each session in about eight minutes.

### Measures and analyses

The overall measure of performance for each game session was total targets found (session length was constant), which was determined by the time needed to find and then consume each target. These two components of performance were measured by search time and consumption time. Search time was operationalized as the time elapsed prior to any agent detecting the target, and consumption time was defined as the amount of time it took to fully consume a given target (maximum of 500 ticks). Both measures were computed on a per target basis, and then averaged for each session. This aggregation created equally sized samples across all conditions for each measurement. Overall performance was also measured by the average total trial time, i.e. the sum of search and consumption time, where lower times corresponded with better performance.

Two factors were manipulated to test efficacy of loose coupling and the role of memory and strategy in collective foraging. The efficacy of loose coupling was tested by comparing different movement rules for autonomous search agents across different sessions, where all agents in each session were governed by the same set of rules. The role of memory and strategy was tested by comparing sessions with and without human intervention, and because simulations are not statistically comparable to humans in terms of their variability, we ran separate analyses with and without human intervention.

To test the outcomes of these measures independently, we conducted separate within-subject analyses of variance (ANOVA) with each as the dependent measure, and two independent variables, human intervention (present or absent) and movement type (random, flocking, distancing, and loose coupling). Any result lower than *p* < 0.05 are considered as statistically reliable. Estimated effect sizes of ANOVA results are represented by partial eta squared (η_p_^2^). Lastly, we also conducted Tukey HSD post-hoc analyses to conservatively test for differences between specific pairs of conditions, even though many of these tests were planned a priori. All remaining figures were generated in R using ggplot2 version 3.3.2^31^.

## Results

### Loose coupling and human intervention promote collective foraging success

We first determined group search performance by assessing the average search time, consumption time, and total targets found in each movement condition with and without intervention.

**Figure 3:**
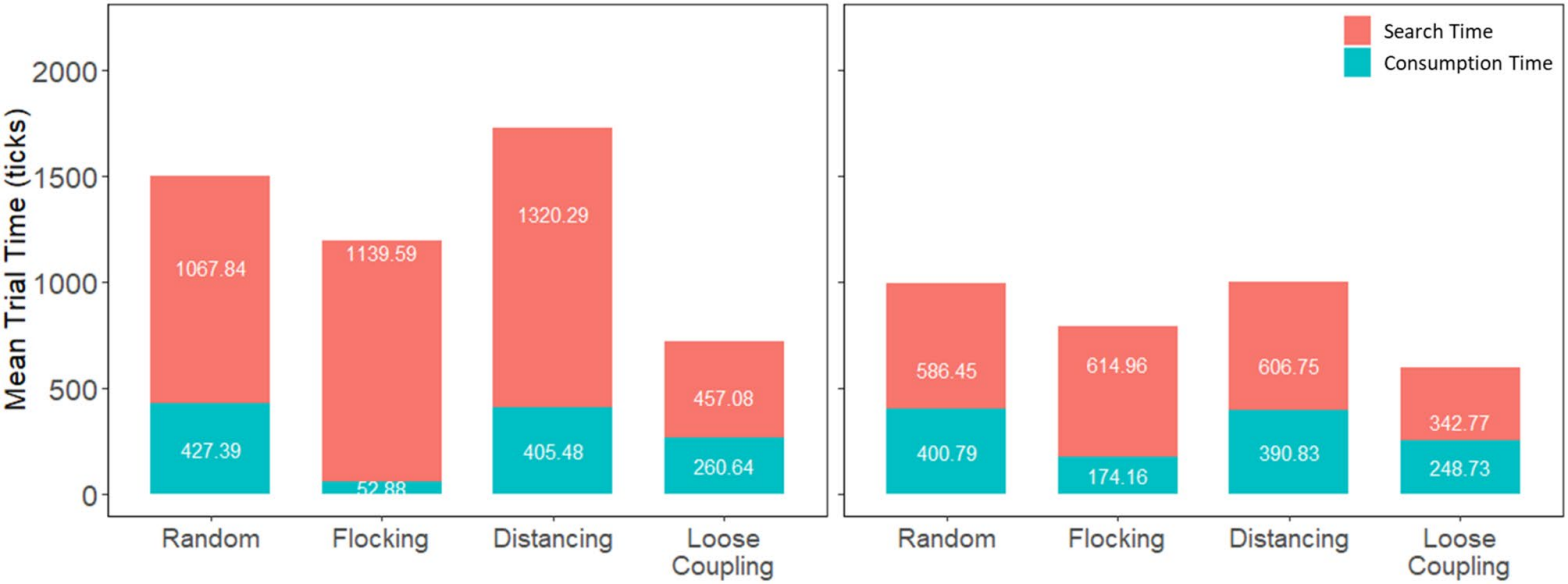
Mean trial time per session as a function of movement type without human intervention (left) and with human intervention (right). Mean trial times are divided into their composite search times (red) and consumption times (teal).

**Figure 4:**
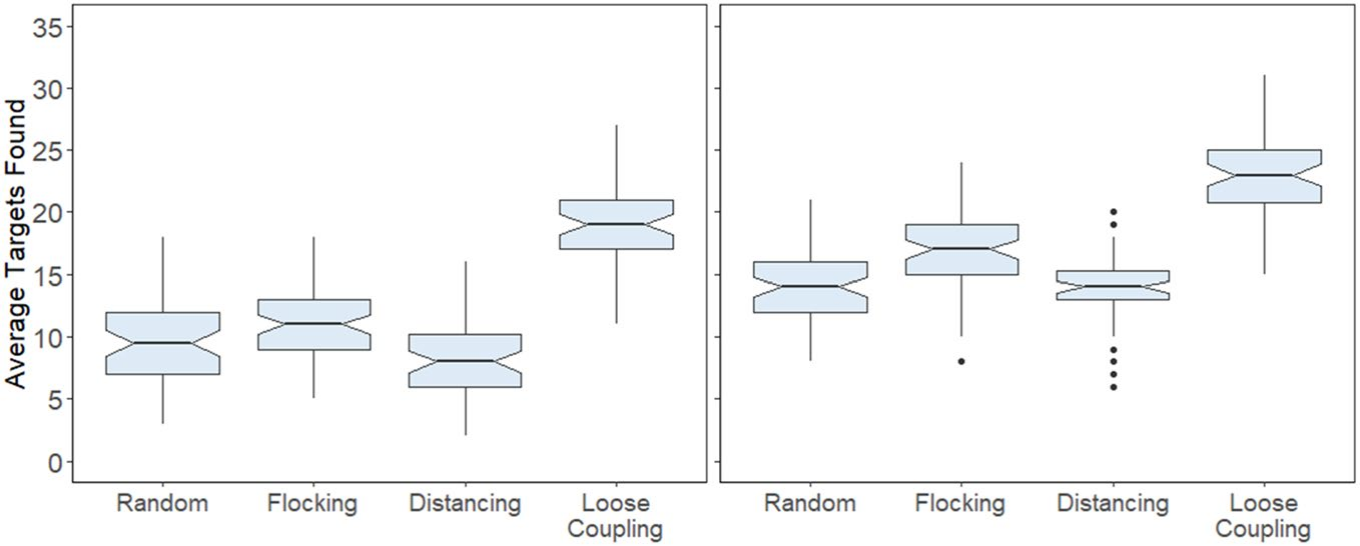
Mean targets found per session as a function of movement type without human intervention (left) and with human intervention (right).

Results showed that search performance as measured by mean trial time was better with loose coupling and human intervention, as seen in the lowest average trial times in Fig. 3. Movement type had a reliable effect on performance without human intervention, *F*(1,59) = 27.65, *p* < 0.001, η_p_^2^ = 0.319, and with human intervention, *F*(1,59) = 20.85, *p* < 0.001, η_p_^2^ = 0.261. The specific direction of effect was supported by post-hoc Tukey HSD comparison tests showing that loose coupling was significantly better than other movement types both with and without human intervention (each *p* < 0.001). By necessity, the same pattern of results was found when performance was measured by number of targets found per session: On average, more targets were found with human intervention (M = 16.94) than without (M = 5.89), and more targets were found with loose coupling (M = 16.26), compared with other movement types (M = 9.8). Human intervention did not interact with movement type, *F*(1,59) = 2.55, *p* = 0.116, η_p_^2^ = 0.141, which indicates that human intervention resulted in more targets found on average per session for all movement conditions (M = 11.04), see Fig. 4 below.

Next we broke the performance measure of mean trial time into its two component parts of search time (i.e. the time from start of a trial to when any of the agents detecting the target) and consumption time (the time from the first agent landing on the target to its complete consumption, which went faster as additional agents landed to share in consumption). Mirroring mean trial times, search times were fastest in the loose coupling condition regardless of human intervention: without, *F*(1,59) = 48.17, *p* < 0.001, η_p_^2^ = 0.449, and with intervention, *F*(1,59) = 41.24, *p* < 0.001, η_p_^2^ = 0.411, refer back to Fig. 3. Tukey HSD for both loose coupling conditions were significantly faster than all other respective movement conditions, *p* < 0.001. By contrast, consumption times were fastest in the flocking condition: without, *F*(1,59) = 542.2, *p* < 0.001, η_p_^2^ = 0.902, and with intervention, *F*(1,59) = 56.79, *p* < 0.001, η_p_^2^ = 0.49. All Tukey HSD flocking condition comparisons were significant, *p* < 0.001. Flocking produced faster consumption times because agents were usually clumped together when the target was found, so they all landed on the target to consume it together. This effect of flocking was predicted to occur, and we also predicted that the distancing condition would produce the fastest search times by means of a divide and conquer strategy. Results were not consistent with this latter prediction because adding flocking to distancing actually improved search times. We return to this unexpected result later when we present analyses of the rate at which agents collectively covered the search area.

Analyses of search times and consumption times as a function of human intervention found that, again mirroring mean trial times, human intervention improved search times substantially across all four movement conditions, albeit less reliably for loose coupling because of an apparent ceiling effect (loose coupling without human intervention already produced fast search times): *F*(1,59) = 22.24, *p* < 0.001, η_p_^2^ = 0.086. By contrast, human intervention improved consumption times in most conditions, *F*(1,59) = 80.00, *p* < 0.001, η_p_^2^ = 0.253, but surprisingly, humans caused slower consumption rates in the flocking condition, comparison Tukey HSD *p* < 0.001. The apparent detriment of human intervention on flocking consumption times can be explained by humans finding targets on their own, without the benefit of other agents nearby to join in consumption. This explanation is further addressed in the next section.

### Loose coupling diversifies groupings of search agents

Collective foraging performance was best with loose coupling, which was predicted based on the hypothesis that loose coupling balances the benefit of flocking versus distancing. This balance should result in more flexibility in agent groups as they merge and split over time—the agents only partially affect each other’s movements, thereby allowing interactions between search agents to vary as they come in and out of view of each other. To quantify flexibility in grouping, we examined the distribution of numbers of agents in view for each given tick, trial, and agent. If groupings do not change much within each trial, then there should be little variation in the numbers of agents in view, and the distribution should have a sharp peak. By contrast, if groupings vary during a trial, then the numbers of agents in view should vary, and hence their distribution should be more spread out.

We used Shannon entropy to quantify the degree to which the frequency distributions in groupings were more peaked (low entropy) versus more spread out (high entropy). Entropy has been used previously for capturing the fission–fusion dynamics for various groups animal species^32^. Hereafter, we refer to it as *Grouping entropy* which was calculated as,$-\sum [p({\mathrm{x}}_{i})\mathrm{ log}(p({\mathrm{x}}_{i}))]$ where x_*i*_ is the number of agents viewed by $i$-th agent over time, and *p* is the probability associated with the proportion of time that x_*i*_ agents were in view.

To focus on grouping entropy from the perspective of autonomous agents, we removed the human player from entropy calculations, and to make analyses comparable, we removed a simulated agent at random in sessions without intervention so that entropy was computed over zero to eight possible agents in view in both conditions. The first 14 ticks at the start of each new trial (when each new target was generated) was removed to avoid initial transients due to agents starting together from the previous target location. Entropy was computed over the subsequent ticks for each trial, up to the tick when the next target was detected by one of the agents. We also computed grouping entropy with respect to the human agent, and again we removed one autonomous agent at random so that entropy was computed over zero to eight possible agents in view.

**Figure 5:**
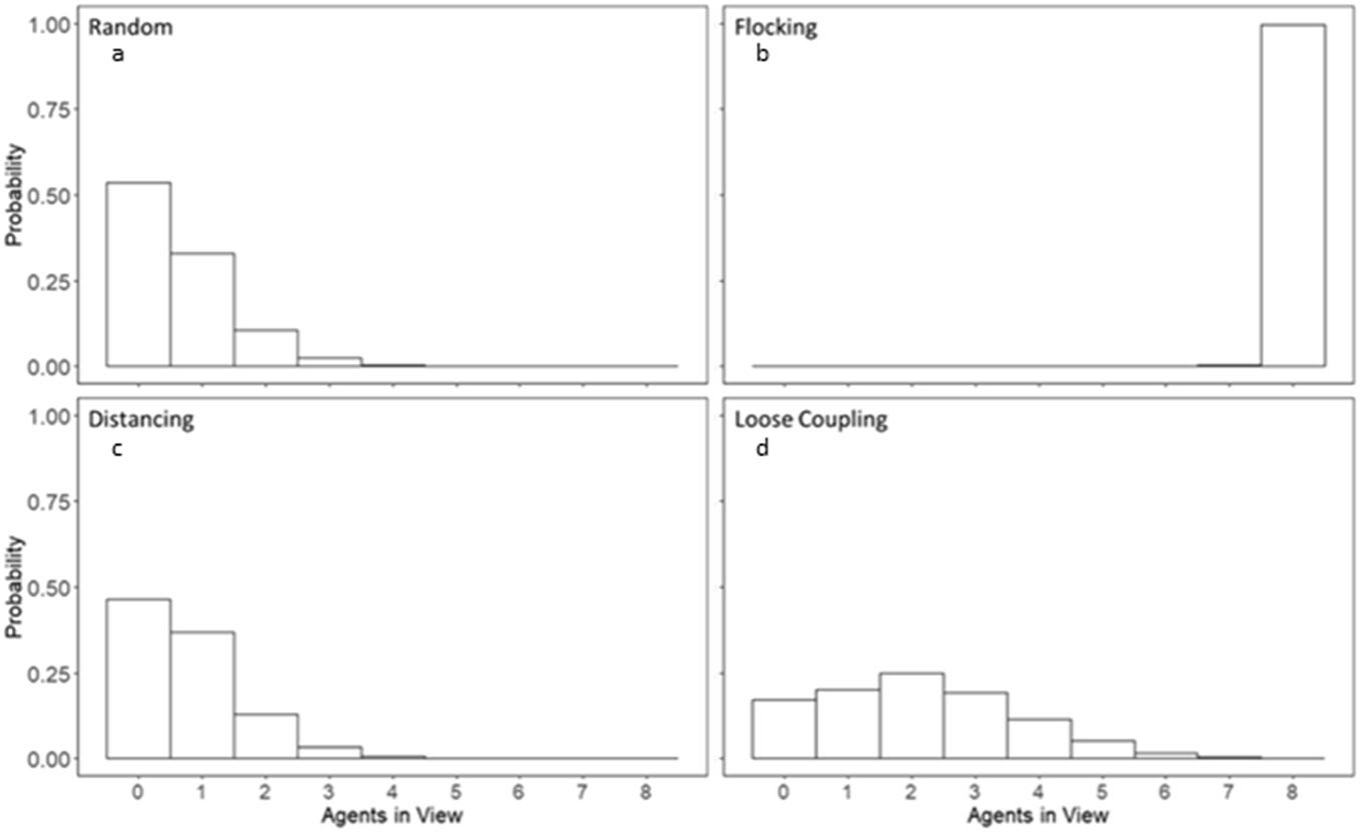
Normalized histogram of the number of agents in view during the search time period respective to one autonomous agent without intervention.

**Figure 6:**
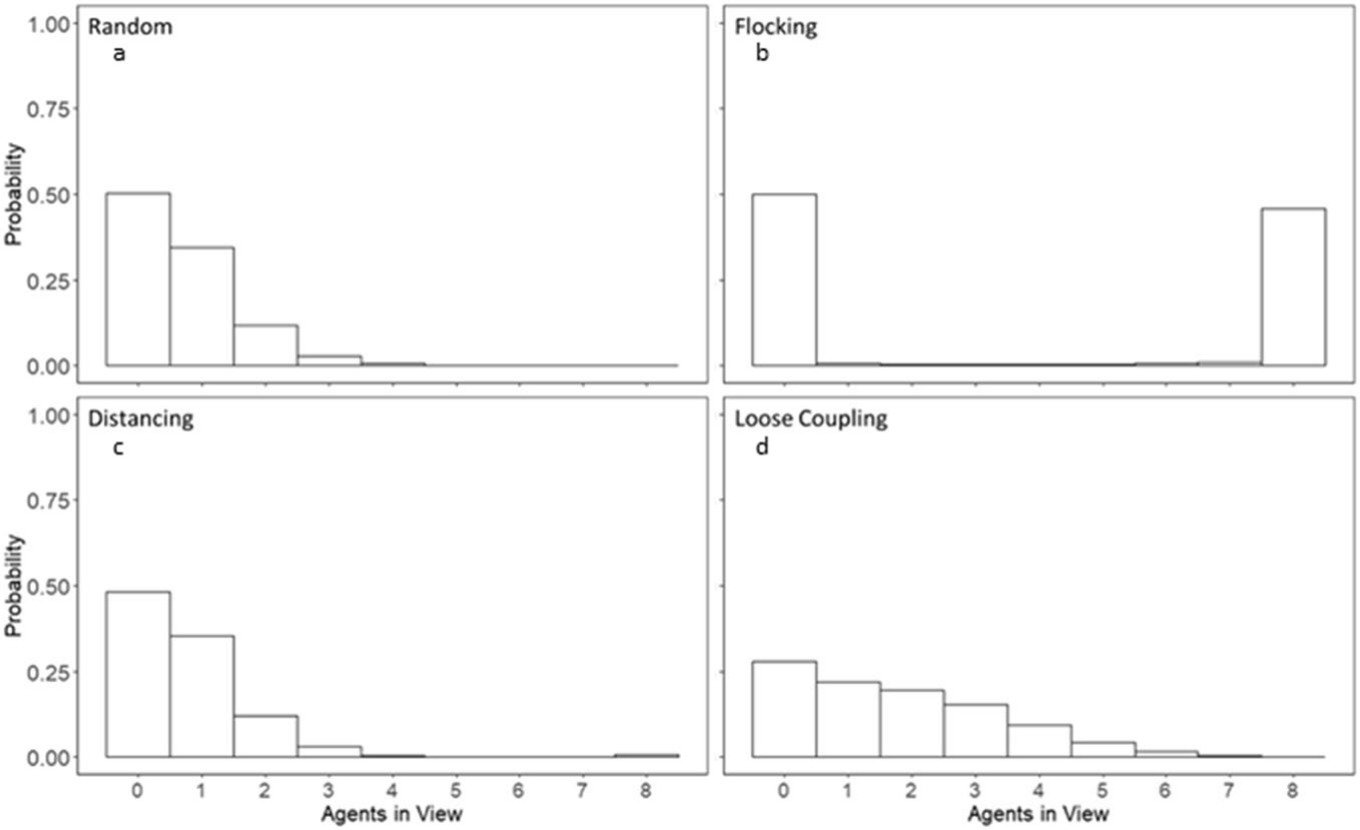
Normalized histogram of the number of agents in view during the search time period respective to only human agents.

**Figure 7:**
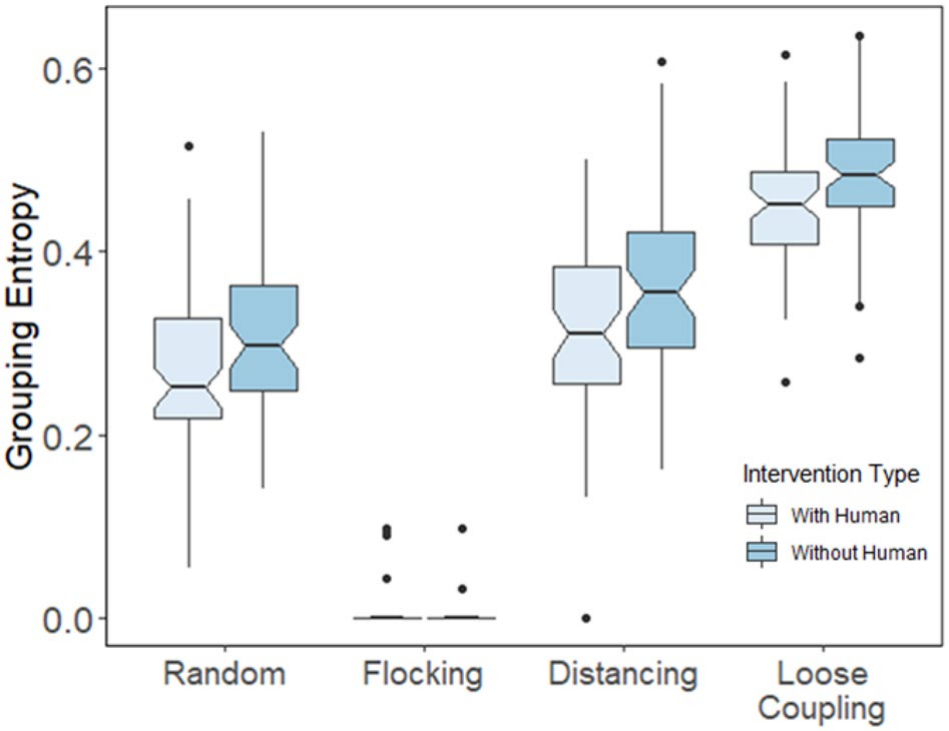
Entropy values for simulated agents as a function of movement type (Random, Flocking, Distancing, and Loose coupling) in the experiment (with human) versus the simulation (without human).

**Figure 8:**
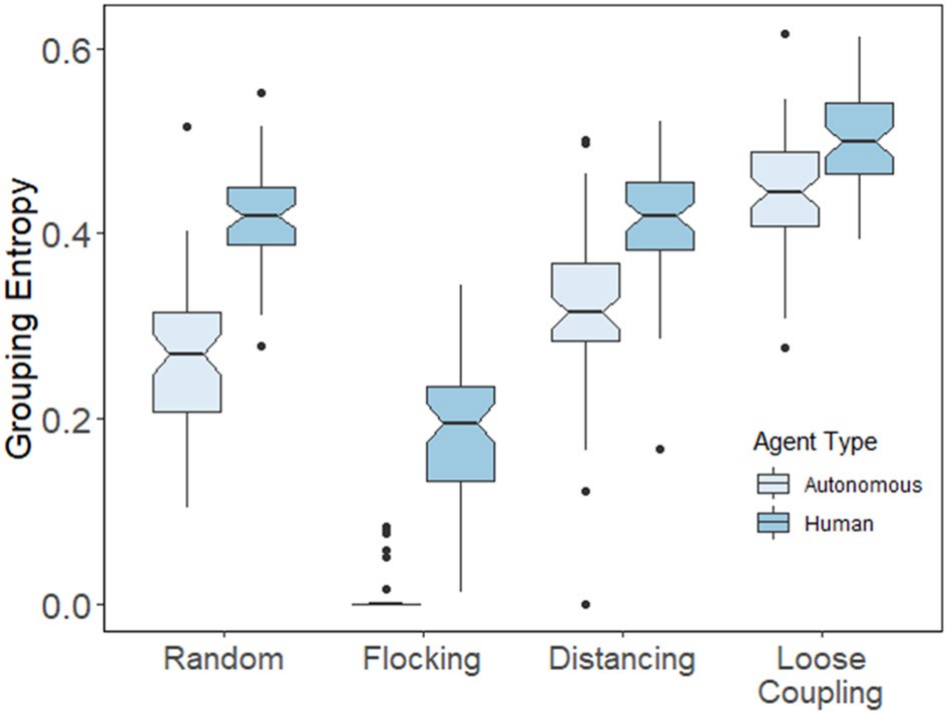
Entropy values by movement condition (Random, Flocking, Distancing, and Loose coupling) for human players (Human) versus simulated agents (autonomous) in the experiment.

Figures 5 and 6 show the proportion of agents in view aggregated over trials and individuals for each movement condition with respect to autonomous agents in the simulation (Fig. 7) and with respect to human agents in the experiment (Fig. 8). These histograms show that the rules governing agent movements and interactions had large effects on agent groupings. The random and distancing conditions were similar in that agents traveled solo much of the time, with another agent in view sometimes, and two or three more on occasion. Adding the flocking rule to each of these two conditions resulted in opposite effects on grouping entropy than the other movement conditions. Flocking agents constantly maintained each other within their respective fields of view, best shown in Fig. 5b. Flocking plus correlated noise (the random condition) resulted in all agents converging and moving together such that variability caused by noise was not enough to disperse the single grouping of autonomous agents once it was formed. By contrast, adding the distancing term to flocking (along with the correlated noise) was sufficient to counteract flocking and disperse agents such that their flight configurations varied over time. This variation resulted in more varied group sizes and hence more variability and greater entropy in the numbers of agents in view.

We tested the effects of movement type and human intervention on grouping entropy using two different statistical analyses. First, we tested entropy values for individual simulated agents with and without human intervention, as a function of movement type, as shown in Fig. 7. Entropy values were minimal in the flocking condition because agents were always in a unified group, so we removed this condition from statistical analyses. Second, we ran the same analyses after removing trials in the human intervention condition when the human player was first to find the target, so that entropy values were not directly affected by human intervention. The second analysis allowed us to ascribe differences to the effect of human intervention on the movements of autonomous agents.

We conducted a mixed-effects ANOVA with movement condition as a within-subjects factor, human intervention as a between-subjects factor, and entropy as the dependent variable. First there was a significant main effect of human intervention whereby human players caused autonomous agents to exhibit less entropy in their distributions over agents in view, *F*(1,59) = 21.43, *p* < 0.001, η_p_^2^ = 0.058; and a marginally significant main effect of movement type, *F*(2,59) = 2.41, *p* = 0.091, η_p_^2^ = 0.014. The interaction was non-significant, *F*(2,59) = 0.49, *p* = 0.613, η_p_^2^ = 0.003. Individual post-hoc tests confirmed that grouping entropy was highest with loose coupling compared with the distancing and random conditions, *p* < 0.001. Human intervention appeared to decrease grouping entropy for autonomous agents by giving them less time to group by means of converging on targets. This decrease in grouping entropy was evident even in the random condition when humans had no direct effect on agent movements—instead, humans had indirect effects because they helped find and consume targets more quickly, thereby decreasing the time available for agents to converge on targets, leaving them less grouped and more disbursed in general.

In our second analysis, we compared the same grouping entropy measure as before but for human players against grouping entropy for individual simulated agents in the experiment with human intervention (Fig. 8). We ran another ANOVA like the previous analysis, but with intervention type replaced by agent type (human or autonomous) as a between-subjects factor, again excluding the flocking condition from movement type. We found that grouping entropy was greater for humans compared with autonomous agents, *F*(1,59) = 213.85, *p* < 0.001, η_p_^2^ = 0.379, and grouping entropy was again influenced by the movement condition, *F*(2,59) = 8.11, *p* < 0.001, η_p_^2^ = 0.044, with post-hoc tests showing that entropy was greatest with loose coupling, *p* < 0.001. There was also an interaction such that grouping entropy for human players was more like autonomous agents when the latter were loosely coupled compared with other movement types, *F*(2,59) = 16.9, *p* < 0.001, η_p_^2^ = 0.088. Moreover, human movements exhibited the most grouping entropy when coordinating with loosely coupled agents based on our post-hoc comparisons, *p* < 0.001.

In summary, grouping entropy was higher, and performance was better, with loose coupling overall, and with human intervention overall (human intervention lowered entropy for simulated agents, but only as a byproduct of shortening time for them to converge on targets). We infer from the main pattern of results that collective foraging in our simulation benefits from loose coupling between autonomous agents as well as between agents and humans.

### Human intervention benefits search performance for non-random agents

Entropy analyses in the previous section showed that human intervention decreased the grouping entropy of autonomous agents, even though performance was generally better with human intervention and with increased grouping entropy. Therefore, it is unclear whether human intervention improved the way that autonomous agents searched, or if humans are simply better searchers and therefore find and consume more targets than autonomous agents.

To test the search performance of autonomous agents themselves, we measured how fast they covered the game space when searching for each next target, and we compared their rates of search area coverage with and without human intervention as a function of movement type. Specifically, *area search rate* was computed as the number of unique pixels searched on each trial, divided by the time spent searching prior to finding the target, and converted into a percentage of total pixels (200 × 200 = 40,000 pixels).

**Figure 9:**
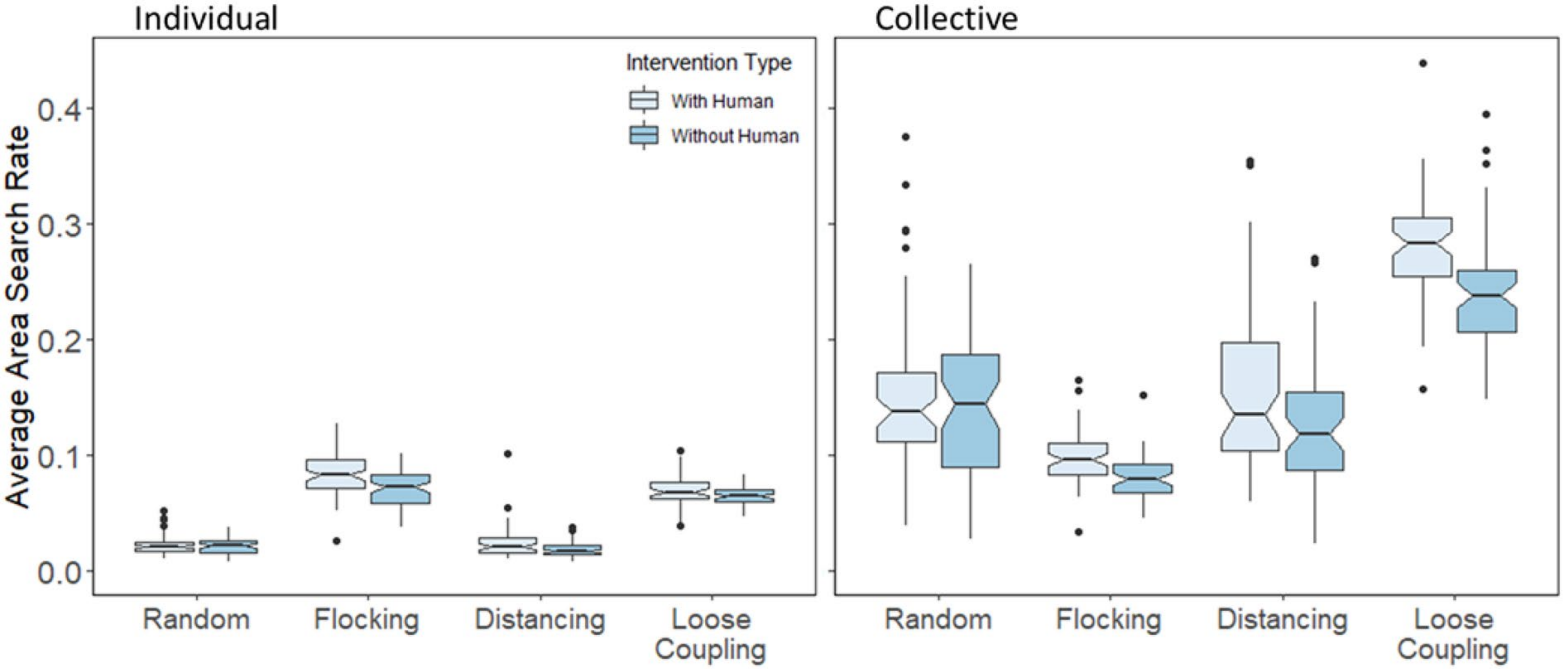
Area search rate for autonomous agents, averaged for each session, and plotted as a function of movement type and human intervention for agents individually (left) and collectively (right).

To test more specifically how human intervention affected autonomous agents, we measured area search rate at both the individual and collective levels for autonomous search agents. For the individual level, search rate was computed per agent and then averaged to gauge how fast each agent covered space separately, whereas for the collective level, search rate was computed for all agents simultaneously to gauge how fast the group covered space collectively. Figure 9 shows area search rates for autonomous agents with and without human intervention (the human is always removed from rate calculations, and as before, trials were excluded when search was terminated by the human player finding the target first), for individual search as well as collective search. We used ANOVA models as in the previous results for grouping entropy, but with area search rate as the dependent measure instead, and flocking was included this time.

We found that human intervention improved both individual search rates and collective search rates, *F*(3,59) = 43.09, *p* < 0.001, η_p_^2^ = 0.044, but had no effect on random movement conditions because humans had no direct effect on random agent search movements, all post-hoc tests *p* > 0.95. The benefit of intervention was greater for collective versus individual area search rates, *F*(3,59) = 17.28, *p* < 0.001, η_p_^2^ = 0.018, indicating that human intervention reduced overlap in autonomous agent search areas, as well as reduced the degree to which individual agents returned to areas they already covered. Conducting a post-hoc Tukey HSD analysis found that individual area search rates were not reliably different between the flocking and loose coupling conditions, all *p* > 0.7, but collective search rates were greater with loose coupling compared with flocking, all *p* < 0.001. These results indicate that loose coupling preserved the individual diffusiveness of flocking agents, whose individual grouping entropy was relatively high. By contrast, the addition of distancing helped to reduce agent overlap—collective grouping entropy was highest for loose coupling—and thereby improve collective search performance.

### Human search benefits from coordinating with loosely coupled agents

The previous section focused on the beneficial effect of human intervention on the individual and collective search performance of autonomous agents as a function of different movement rules. We can also test whether different movement rules have different effects on human search performance. In theory, human players could search on their own, unresponsive to the movements of other agents. However, to the extent that players try to guide or otherwise coordinate with autonomous agents, the efficacy of human search movements may be affected by the way agents move and coordinate. Results presented earlier showed that human intervention affected autonomous agents via their grouping entropy, and agents affected human players in kind. Given that human players showed the greatest grouping entropy when agents themselves showed the greatest grouping entropy in the loose coupling condition, we can hypothesize that human search performance may benefit from coordination with loosely coupled agents.

**Figure 10:**
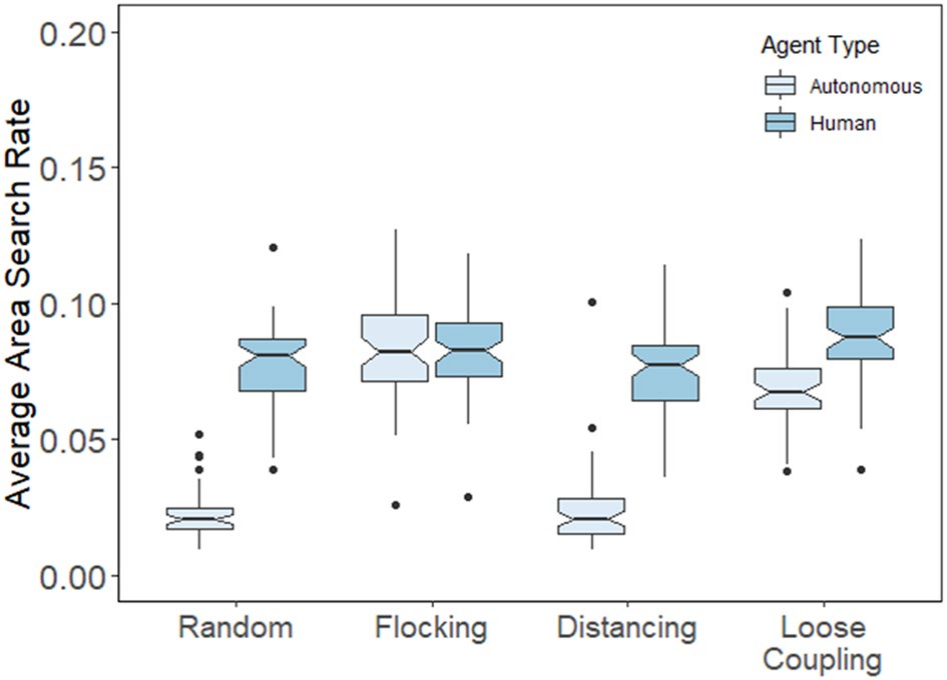
Area search rate averaged for each session and plotted as a function of movement type and agent type for human players and individual agents with intervention.

To test the effect of movement rules on human search performance, we computed area search rates for the human players individually. Not surprisingly, as shown in Fig. 10, humans overall covered the search space at a faster rate than the individual agents they foraged with, *F*(1,59) = 536.04, *p* < 0.001, η_p_^2^ = 0.534. The exception to this overall effect was in the flocking condition where humans searched at about the same rate as agents they were coordinating with. Individual area search rates were relatively high for flocking agents because maintaining a single, steady bearing is a reasonably good strategy for covering a space with periodic boundary conditions.

We also found marginal differences in human area search rates depending on the movement rules governing autonomous agents, *F*(3,59) = 2.57, *p* = 0.055, *η*_p_^2^ = 0.032. As predicted, a post-hoc analysis showed human search performance was better with loosely coupled agents compared with distancing (*p* < 0.001) and random (*p* < 0.01) movement conditions. Although no other comparisons were reliable, Fig. 10 shows that human search rates were also elevated when coordinating with flocking agents, which may be attributable to humans sometimes following the flock as they search at a faster rate than agents in the random and distancing conditions.

Taken together with results from the previous section, we can conclude that human players and loosely coupled agents benefitted from each other to improve search performance by virtue of flexibly coordinated movement patterns, as evidenced by higher area search rates along with higher values of grouping entropy.

### Human players adapt their foraging strategies to agent behaviors

To this point, we have interpreted the result that human players improve collective foraging by means of memory and strategy, but the evidence has not been direct. It is difficult to infer specific strategies from game play data alone, but one apparent choice that players can make in collective foraging is the emphasis on finding versus consuming targets. Players may try to find targets with other agents following or not, or they may instead seek out other agents to collectively consume each target so the next one comes faster. Human players may improve collective foraging in part by adapting their emphasis on finding versus consuming targets based on the rules governing movements of autonomous agents.

To measure the emphasis on finding versus consuming targets, we analyzed the proportion of targets found versus consumed by human players as a function of movement type. As a baseline, if humans are no better than their autonomous counterparts, then they should find targets 10% of the time (0.1 proportion of times) and consume 10% of the target units (recall that each target consisted of 500 consumption units), given that the human player is one of ten foraging agents. The difference between finding and consuming proportions is a measure of the emphasis that human players placed on one versus the other component of collective foraging.

**Figure 11:**
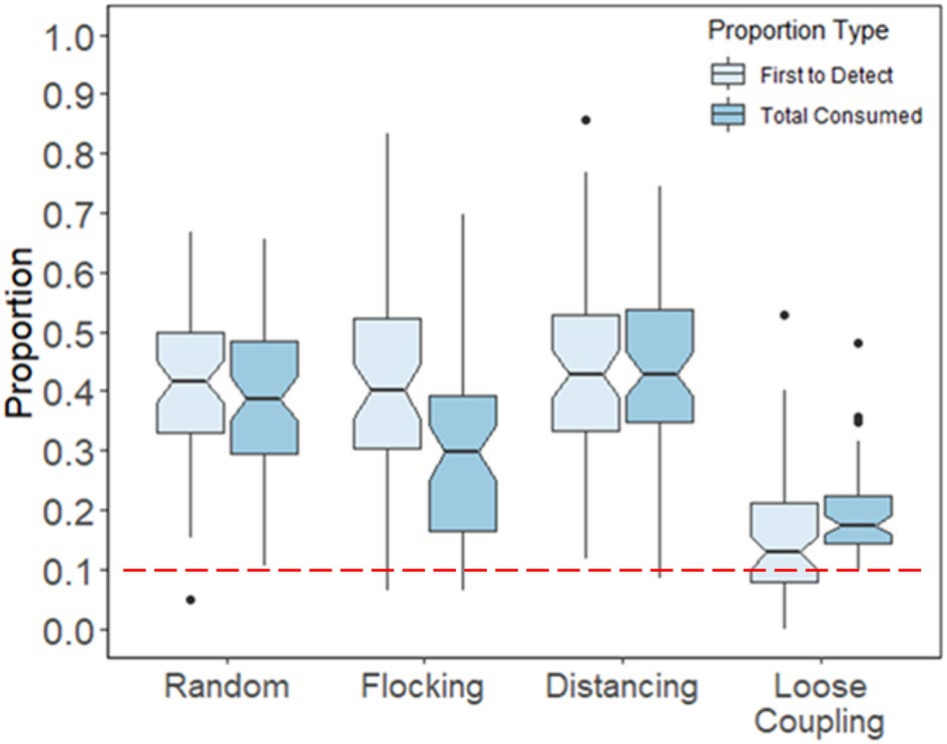
Finding and consuming proportions for human players as a function of movement type. The red line indicates the expected proportion to be found and consumed if human performance was no better than that of simulated agents. Proportions above the expected baseline indicate the degree to which humans outperformed simulated agents.

Figure 11 plots the two proportions for human players as a function of movement type. First one can see that both proportions were significantly above 0.1 in all movement conditions, all post-hoc tests *p* < 0.001. Greater-than-chance proportions are evidence that at least some benefit of human intervention for collective foraging comes from the superiority of human players, in that they both find and consume more targets than autonomous agents. Post-hoc analysis showed this benefit was reliably less in when agents were loosely coupled compared with other movement types, all *p* < 0.001, because loose coupling was the most effective movement rule.

Regarding adaptations in strategy, Fig. 11 indicates that the differential between proportions varied as a function of movement type, *F*(3,59) = 5.74, *p* < 0.001, *η*_p_^2^ = 0.035. Specifically, players emphasized consuming targets over finding targets when coordinating with loosely coupled agents, *t*(59) = 4.96, *p* < 0.001, whereas players did the opposite when coordinating with flocking agents, *t*(59) = − 11.43, *p* < 0.001. This contrast is evidence that players adapted their strategy to the movement rules for agents—it was more beneficial for players to help with search when the collective area search rate was relatively low in the flocking condition, whereas it was more beneficial for players to help with consumption when collective area search rate was relatively high in the loose coupling condition.

## Discussion

The main goal of this study was to investigate the benefit of balancing individual and collective modes of interaction to succeed in social foraging. We designed an agent-based model in which finding targets benefitted from individual agents diffusing across the search space, whereas consuming targets benefitted from convergence of agents on target locations. We formulated movement rules that balanced a tendency towards distancing with a tendency towards flocking, and simulations showed that loose coupling among agents was beneficial to group foraging performance. Analyses of grouping entropy showed that loose coupling diversified the range of more individuals to more collective search configurations because flocks of various sizes formed and dissolved as search unfolded.

Our simulation results show that loose coupling is beneficial even when agents are memoryless and unable to learn, adapt, or develop strategies through experience. The diversity of search patterns did not come from decision-making of any kind—it was instead driven primarily by injecting noise with the CRW movement component, plus additional randomness from positioning of targets. Our simple model of loose coupling is useful in its economy of mechanism and may be appropriate for collections of simple organisms and artificial agents with minimal capacity for computation. However, foraging is necessary for the survival of all mobile species, including for humans and other social animals with extensive capacities of memory, learning, and strategy to drive decision-making as foraging unfolds. To test how decision-making capacities might interact with simple rules of loose coupling, we compared simulations of autonomous agents with and without human intervention.

Human players exhibited an even greater diversity of search configurations than loosely coupled agents, and both humans and autonomous agents covered the search area at faster rates in the loose coupling condition compared with too much distancing or too much flocking. These results provide evidence that abilities like learning and memory may complement simpler rules of loose coupling to support social foraging, rather than supplant them. It is difficult to determine whether movements were controlled based on strategies learned through experience, but we did find evidence that players adapted their strategies to coordinate with agents differently depending on their search behaviors. Human players emphasized finding and consuming targets individually when agents distanced too much or flocked too much, whereas they coordinated more with simulated agents were loosely coupled. This finding suggests that intelligent agents can learn to leverage other agents for the good of the group, depending on their abilities and performance. Humans varied in performance and behavior from one player to the next (individual differences are universal to human behavior), but more information is needed about the backgrounds and intentions of human players to understand these individual differences.

Our agent-based model proved useful for demonstrating the benefits of loose coupling and adaptive foraging strategies, but future studies could undertake more thorough analyses of the model and its parameters to understand which aspects are most important for loose coupling in social foraging. Also, while the distancing and flocking rules combined to produce loose coupling, they were not quite complementary on their own. Flocking had the desired effect of longer search times offset by shorter consumption times, relative to random search, but distancing did not have the reverse effect—instead, distancing did not have an appreciable effect beyond noise from the CRW rule, although it synergized strongly with the flocking rule. Future studies may consider a different form of distancing that has the opposite effect of flocking, i.e. shorter search times offset by longer consumption times. Future work may also consider implementing measures of grouping and movement coherence^33^. Such measures that help to identify to the effect of movement conditions on the velocity of the whole group.

Finally, it would be informative to study how groups of human foragers coordinate to play our social foraging game. The most salient question is whether players would still exhibit signs of loose coupling in terms of flexibly diverse groupings, and whether group performance would still benefit loose coupling. Theories of self-organization suggest that loose coupling may be generally useful for adapting coordinated behavior to respond to changes in conditions as they unfold^34^. For instance, it may be useful to follow one or more agents when they are first encountered in the hopes of finding targets and consuming them together, but it may become more beneficial to break from the group and seek new search opportunities as time goes by following the group with no success. In this scenario, loose coupling may enable agents to enact a “stay-or-go” decision between exploiting nearby agents or exploring new opportunities, similar to the stay-or-go decision at the heart of optimal foraging theory^35–37^. The agent-based modeling and experimental paradigm introduced herein may be extended to investigate these and other questions about individual and collective foraging.

## Data Availability

Data, Netlogo, and R codes are available upon request.
